# Phenotyping common beans for adaptation to drought

**DOI:** 10.3389/fphys.2013.00035

**Published:** 2013-03-06

**Authors:** Stephen E. Beebe, Idupulapati M. Rao, Matthew W. Blair, Jorge A. Acosta-Gallegos

**Affiliations:** ^1^CIAT—International Center for Tropical AgricultureCali, Colombia; ^2^Formerly of CIAT, CIAT—International Center for Tropical AgricultureCali, Colombia; ^3^Bean Program, INIFAP Research Station, Instituto Nacional de Investigaciones Forestales, Agrícolas y PecuariasCelaya, Mexico

**Keywords:** Phaseolus, field technique, abiotic stress, breeding, stress physiology

## Abstract

Common beans (*Phaseolus vulgaris* L.) originated in the New World and are the grain legume of greatest production for direct human consumption. Common bean production is subject to frequent droughts in highland Mexico, in the Pacific coast of Central America, in northeast Brazil, and in eastern and southern Africa from Ethiopia to South Africa. This article reviews efforts to improve common bean for drought tolerance, referring to genetic diversity for drought response, the physiology of drought tolerance mechanisms, and breeding strategies. Different races of common bean respond differently to drought, with race Durango of highland Mexico being a major source of genes. Sister species of *P. vulgaris* likewise have unique traits, especially *P. acutifolius* which is well adapted to dryland conditions. Diverse sources of tolerance may have different mechanisms of plant response, implying the need for different methods of phenotyping to recognize the relevant traits. Practical considerations of field management are discussed including: trial planning; water management; and field preparation.

## General information

### Importance of beans in the human diet

Common beans (*Phaseolus vulgaris* L) are the most important grain legume for human consumption (Broughton et al., 2003; Beebe, 2012). Given that most protein consumed by the poor is from plant sources, being protein-rich, beans play an especially significant role in the human diet. Although far less important than cereals as a source of calories, beans often supply a significant proportion of carbohydrates [Food and Agriculture Organization of the United Nations (FAO), 2001]. Like other legumes, they are also a key source of minerals, especially iron (Graham et al., 2007).

In Latin American countries, national per capita consumption of beans is typically between 12 and 18 kg per year, but this does not reflect differences in urban versus rural consumption, nor income differences (Broughton et al., 2003). In rural Nicaragua, for example, per capita consumption can be as high as 36 kg per year among the more affluent, whereas the rural poor cannot satisfy their needs and consume about half that amount (FAO, 2001). In Africa, bean consumption can be as high as 60 kg per capita per year in countries like Rwanda or in western Kenya. However, here as well, availability and cost often limit bean consumption and real consumption levels may be lower. In Mexico, an estimated per capita consumption of 12 kg per year of dry beans has been registered, but almost 100,000 tons are transformed into canned beans with a yield of 3.5 kg of canned bean product per kg of dry beans. The need for ready cooked bean products is increasing, due to the inclusion of an increasing number of women in the work force.

### Cultivated area and yield performance under optimal conditions

Total world production cannot be calculated with certainty due to confusion with other legumes in some of the data, but is between 11 and 12 million tons (FAO, 2006). Latin America is the region of greatest production of common beans, representing about 50% of world volume, followed by Africa with 25%. Brazil, Mexico and the United States of America are the three largest producers in the western hemisphere. In Mexico, runner beans (*Phaseolus coccineus* L) are a relatively important crop in the highlands, but data on this species are included with those for common beans.

In Africa, most bean production is found in the eastern and southern highlands, extending from Ethiopia to South Africa, with Kenya being the largest producer in the region. In West Africa, bean production is localized in specific environments, with Cameroon being the principal producer. Beans are a minor crop in Europe and North Africa, concentrated around the Mediterranean, in Spain, Italy, Morocco, Algeria, and the Balkan states. In Asia, beans are spread in an extensive band from Turkey through Iran and the Himalayan foothills, and east through Myanmar and China. India is cited as a major producer of common beans (FAO, 2006), but these figures undoubtedly include other legumes (Singh, 1999).

Beans are traditionally a small farmer crop, often grown in complex farming systems in association or rotation with maize, sorghum, bananas, or other crops (Broughton et al., 2003). The range of growth habits (from determinate bush types to vigorous climbers), and the range of growth cycles (from 2 to 10 months in length) make beans a crop that fits many production niches. Nevertheless, beans are becoming increasingly commercial with the trends of urbanization and market globalization. These trends impact on both small farmers who market excess production to local urban centers, and large commercial farmers in Argentina, China, Mexico, the USA, and Canada with an eye to export markets. Small farmers are also organizing themselves to tap into opportunities to export in countries like Bolivia, Ethiopia, Nicaragua, and Peru, each of which report from US$20–100 million in bean exports annually.

Experimental yields of bush beans can be 4 t ha^−1^ or more, while climbing beans can reach 6 t ha^−1^ under a trellis system. On-farm yields, as expected, are far below experimental yields. National averages in Latin America range from 600 to 950 kg ha^−1^, with a long-term tendency to increase, while national averages in Africa are similar but tending to decline (FAO, 2006). Total production in both regions is increasing. This trend in Latin America is driven by increasing yields on a stable total area, whereas in Africa, increased production reflects an increased area planted despite declining yields. Anecdotal reports suggest that declining yields in Africa are due to the extension of the crop into marginal production areas with poorer soil fertility and/or drought (Buruchara, pers. communication). This highlights the importance of attention to abiotic stress resistance, including water deficits, which will be emphasized in this review.

As expected, countries with technified agricultural systems present much higher yields than tropical and developing countries. In the USA, average yields in the past decade range from 1.64 to 1.96 t ha^−1^ (USDA, 2007), albeit with significant regional differences. Similarly, average yields in Argentina and Colombia are about 1.2 t ha^−1^ due to varietal selection, and in Brazil under intensive management and irrigation, yields average 1.8 t ha^−1^ (Broughton et al., 2003). Although well above yields in most developing countries, these are still as much as 3 t ha^−1^ below the yield potential of the crop. While it is clear that the yield gap is a generalized phenomenon, yields in drought-endemic regions are typically lower. The largest single drought susceptible production area in the world is in highland Mexico, where more than a million hectares of beans are cultivated, and where yields fall below 0.4 t ha^−1^ in dry years. In Northeastern Brazil, which accounts for another million hectares, yields are around 0.45 t ha^−1^, which is far lower than the 1.5 t ha^−1^ obtained in the more developed southern state of São Paulo (Conab, 2007).

Low yields are undoubtedly due in part to the direct effect of droughts, and in part to the fact that dry areas are also poverty hot spots where there is less capital investment. The Pacific coast of Central America, where most of the population lives, is another drought-prone region, as are Haiti and eastern Cuba in the Caribbean. In Africa, an estimated 682,000 hectares of beans are cultivated in semi-arid environments, with annual yield losses to drought of 781,000 tons across all environments (Wortmann et al., 1998). The drought-endemic area stretches from eastern and central Ethiopia, south through eastern Kenya and the Rift Valley, and through northern Tanzania. Kenya has the largest area of beans under threat of drought, often resulting from failure of the short rains. Occasionally, severe droughts also occur in Southern Africa, affecting Malawi, Zimbabwe, and Mozambique. They are often associated with “El Niño” weather events. Although drought may be less frequent than in eastern Africa, southern Africa enjoys only one rainfed cropping season per year. Therefore, droughts in this region have an especially tragic impact. Moreover, climate models predict that this region will become drier with global climate change (Williams et al., 2007).

### Genetic and genomic resources

The genetic structure of common beans has been reviewed frequently (Gepts and Debouck, 1991; Singh et al., 1991; Broughton et al., 2003), and is only summarized here. Cultivated common beans display a well-defined genepool structure that originates in the wild bean ancestor. Wild common beans grow as a viny annual herbaceous plant in a sub-humid premontane forest ecology from northern Mexico to northern Argentina (Toro et al., 1990). Genetic analysis using amplified fragment length polymorphism (AFLP) reveals at least four wild bean genepools, centered in: (1) Middle America (Mexico and Central America); (2) Colombia; (3) western Ecuador and northern Peru; and (4) the southern Andes (Tohme et al., 1996). Cultivated bean genepools derive principally from the Middle American wild bean pool, and the southern Andean pool. Additionally some incipient domestication or introgression appears to have occurred in Colombia (Gepts and Bliss, 1986; Chacón et al., 1996; Islam et al., 2001b).

Wild common bean populations are under threat due to urbanization and intensive cattle grazing, and they occur disproportionately in regions where climate change will impact on natural ecosystems (Williams et al., 2007). The Andean and Middle American genepools of cultivated beans are distinguished clearly by DNA markers (for example, Beebe et al., 2000; Islam et al., 2004; Blair et al., 2006a), by plant and seed morphology (Singh et al., 1991), by reaction to diseases including anthracnose (Mahuku et al., 2003) and angular leaf spot (Mahuku et al., 2002), and by grain mineral content (Islam et al., 2001a), among other traits.

The major genepools in turn have been divided into races based on plant morphology, adaptation range and agronomic traits. The Middle American genepool was divided into the races Durango (prostrate bush types with medium-sized seed from dry highland Mexico), Jalisco (climbing beans from the moist highlands of central Mexico), and Mesoamerica (small seeded types, mostly bush habits, from lowland Central America and Mexico; Singh et al., 1991). Beebe et al. (2000) suggested the existence of a fourth race—Guatemala (mostly climbing beans from Guatemala and southern Mexico)—as well as some systematic variation within races. Chacón et al. (2005) found that the races Durango and Jalisco shared a common chloroplast DNA pattern, while the races Guatemala and Mesoamerica each presented a distinctive pattern, emphasizing their evolutionary uniqueness. Díaz and Blair (2006) found a dichotomous structure in the Middle American genepool, with grouping of the Durango and Jalisco races apart from the race Mesoamerica, and novel diversity in some climbing bean accessions potentially from the race Guatemala.

Three races have also been proposed within the Andean genepool (Singh et al., 1991), but their differentiation by restriction fragment length polymorphism (RFLP) or random amplified polymorphic DNA (RAPD) markers is not as clear as in the Middle American genepool (Becerra Velásquez and Gepts, 1994; Beebe et al., 2001). All such races display a common chloroplast DNA composition, suggesting that a single population might have been domesticated (Chacón et al., 2005). Andean races can, however, be distinguished by microsatellite alleles (Blair et al., 2007), and are known to be different in terms of growth habit prevalence and adaptation ranges. They include the races Peru (predominantly highland climbing beans), Nueva Granada (mostly bush beans with mid-altitude adaptation), and Chile (prostrate bush or weak climbers, with temperate adaptation to higher latitudes). Significant introgression has occurred into the Andean genepool from Middle American types that have filtered into the northern Andes since pre-Colombian times (Islam et al., 2004; Blair et al., 2007). Cultivars of the Andean genepool are distinguished by more attractive colors and by larger seed (35–50 g 100 seed^−1^, compared to 20 g 100 seed^−1^ for Mesoamerican beans or 30 g 100 seed^−1^ for Durango types; Singh et al., 1991). A unique white bean cultivated in Spain, the Fabada bean, has grain as large as 100 g 100 seed^−1^! These traits add value to Andean beans and make them an attractive crop for sale. In regions of Africa in which both Andean and Middle American beans are grown, many market-oriented farmers would prefer Andean types, whereas small seeded Mesoamerican types might be used for home consumption.

Over the past decade and a half, the genepool structure and the concept of races within genepools has become the intellectual framework for the improvement of common beans. Breeders now routinely speak of commercial grain classes within the context of races. It is appreciated that crosses among genotypes of the Andean and Middle American genepools represent wide crosses with a low probability of success. There is a sense of which parental types can combine more readily to produce useful progeny. There is a structure within which to explore genetic diversity systematically for useful traits. This represents a significant systematization of knowledge about bean genetic resources accompanied by practical application. Unanswered questions regarding the genetic structure of common beans and its significance include: the extent and potential value of wild and weedy types from Colombia (Beebe et al., 1997); the existence of other possibly unique wild bean populations in Nicaragua, Venezuela, and/or parts of Peru; the origin and potential value of Brazilian landraces of the Andean genepool (Beebe et al., 2001); and the origins of the races, especially Chile and Guatemala (Díaz and Blair, 2006; Blair et al., 2007). A deeper understanding of genetic diversity and population structure within cultivated common beans is necessary both for greater utilization of useful germplasm but also for association mapping of valuable traits such as drought tolerance.

In addition to wild ancestors in the primary genepool, common beans enjoy an extensive secondary genepool that can be crossed quite readily with *P. vulgaris*. Singh (2001) has reviewed the use of these genetic resources extensively. Briefly, two other cultivated species, runner beans (*P. coccineus)* and year-long beans (*Phaseolus dumosus*; = *polyanthus*) are found in this genepool, as well as wild species such as *Phaseolus costaricensis*. All three species are vigorous vines with perennial or semi-perennial tendencies, and are found in moist environments. Wild or escaped types experience intense competition from surrounding vegetation, making aggressive vegetative growth necessary for survival. Of these species, runner beans display wider genetic variability, even at the level of the chloroplast (Tovar, 2001). Runner beans and year-long beans have both been employed as sources of resistance to a wide array of bean pathogens (Singh, 2001), although their use for other traits has been very limited. Some accessions of *P. coccineus* are very tolerant of aluminum toxicity in the field and in greenhouse hydroponic systems [Centro International de Agricultura Tropical (CIAT), 2005; Butare et al., 2011]. Field observations and subsequent greenhouse studies of root systems have revealed that runner beans have thick roots that might have a better potential to penetrate compacted soil than common beans. These are traits that could well contribute to drought resistance, and merit further investigation.

Tepary beans (*Phaseolus acutifolius*) are a fourth domesticated species of the genus pertaining to the tertiary genepool, and are native to the desert highlands of northwest Mexico and the southwest of the USA. As such, they are extremely resistant to drought, heat and cold (Martinez-Rojo et al., 2007), and have been viewed as a potential source of drought resistance for common beans. Greenhouse studies of tepary bean root systems reveal extremely fine roots that penetrate soil rapidly and branch profusely, offering quick access to limited soil water reserves (Butare et al., 2011). Crosses between common beans and tepary beans have normally been difficult, requiring the use of *P. vulgaris* cytoplasm and embryo rescue to obtain F_1_ plants. In spite of difficulties, tepary beans have been used as a source of resistance for biotic constraints, especially common bacterial blight (Coyne et al., 1963; McElroy, 1985). An innovative breeding method called “congruity backcrossing” involving alternate crossing to common bean and tepary bean parents, has permitted a greater degree of cross compatibility between these two species, possibly by gradually improving chromosome pairing (Haghighi and Ascher, 1988). A modification of this system now permits crossing into *P. acutifolius* cytoplasm (CIAT, 2002b). An evaluation of introgression from the tepary bean genome shows that DNA markers in the tepary bean parent can be transferred to the interspecific progeny (Muñoz et al., 2004). Thus, the introgression of drought resistance might be feasible. Modest levels of drought resistance have already been introgressed from tepary beans into common beans, but not yet at the levels of tepary beans, nor at a levels superior to that available within *P. vulgaris* (CIAT, 2002a). As an alternative, the cloning of genes from tepary beans could lead to wider exploitation.

Lima beans (*P. lunatus*) are the fifth domesticate within the *Phaseolus* genus. Lima beans grow over an even wider range of environments than common beans, since they are very tolerant of heat and edaphic problems. It is tempting to introgress traits from lima beans into common beans. However, efforts to date to cross lima beans with common beans have resulted in no more than totally sterile F_1_ plants (Mok et al., 1978). For the foreseeable future, it will be more productive to view lima beans as crop in its own right, unless genes can be extracted from it through molecular biological techniques.

With the advent of DNA markers in the 1980's, multiple mapping populations were created. A list of 14 such populations has been published, including 10 of recombinant inbred lines (RILs; Broughton et al., 2003). The first two maps based on RFLP, and therefore of wider accessibility, were published in the early 1990's (Vallejos et al., 1992; Nodari et al., 1993). Many subsequent maps were based on RAPD markers. Eventually, a total of five maps were harmonized around a core map (Freyre et al., 1998). A sixth map created at CIAT has, likewise, been cross-referenced with the core map (Blair et al., 2003). Cross-comparable RFLP, RAPD, and microsatellite markers form the basis of these maps. A sizable set of microsatellites has been evaluated for polymorphism across parents of multiple mapping populations, as a means of integrating genetic studies through known map positions (Blair et al., 2006a).

CIAT holds other sets of RILs, several created specifically for drought studies, and others for studies of root structure and function in plant nutrition (Blair et al., 2011). A series of publications on roots and phosphorus nutrition resulted from a population of DOR 364 × G 19833 (Liao et al., 2004; Yan et al., 2004; Beebe et al., 2006a). This population proved to be especially useful to reveal the relationship between the acquisition of soil phosphorus and specific root traits, by demonstrating the association of each with common genomic regions. This methodology can readily be extended to drought resistance traits. Other RIL populations include drought-resistant parents BAT 477, G 21212, ICA Quimbaya, and SEA 5.

However, most maps with ample genome coverage have been based on segregation of crosses between Mesoamerican and Andean genotypes to facilitate abundant DNA polymorphism (Blair et al., 2006a). Many polymorphic markers in these crosses are genepool specific, and do not discriminate between DNA of genotypes from the same genepool or race. Since most genetic improvement is carried out within genepools, such markers are seldom useful for gene tagging, mapping or marker-assisted selection (MAS). Thus, there is still a need for a larger set of simple sequence repeat (SSR) or other markers of similar attributes that will permit better genome coverage of crosses among genetically similar materials. CIAT and partners are currently mining additional genomic and complementary DNA (cDNA)-based SSRs for marker development others are developing SNP platforms.

Genomic and cDNA clones have been useful for marker development and gene mining, and they form the basis for some recent sequencing projects. These include an expressed sequence tag (EST) effort consisting of 22,000 sequences derived from four cDNA libraries made from the Mesoamerican genotype, Negro Jamapa, and one cDNA library made from the Andean genotype, G 19833 (Ramírez et al., 2005). Given that these sequences are from drought-susceptible genotypes and represent 3′ end sequences, one option is to develop more cDNA libraries for drought-tolerant genotypes and obtain a set of full-length and 5′ end sequences. In addition, a bacterial artificial chromosome (BAC) library made for the genotype G 19833 is being end-sequenced as part of a physical mapping project for common beans, which will be a useful source of additional markers and partial gene sequences. Finally, for functional analysis of candidate genes of interest, TILLING populations are being created between CIAT and partners at the University of Geneva and the United States Department of Agriculture (USDA)—Puerto Rico which can be screened for mutations in drought gene pathways.

The approach of using ESTs and functional analysis for gene analysis in legumes was described by VandenBosch and Stacey (2003), and includes discussion of applications to improve common beans for nutritional quality and abiotic stress tolerance. Furthermore, an international network of *Phaseolus* researchers called “Phaseomics” maintains communications among bean scientists. A description of their interests and stocks under development has been published in Broughton et al. (2003) a reference genome sequence will soon be published.

### Relevant results published in the area of drought adaptation

For common beans, the working definition of drought would be the inadequacy of water availability, including precipitation and soil moisture storage capacity, in quantity and distribution during the life cycle of the crop, which restricts the expression of the full genetic potential of the cultivar. Terminal or intermittent drought stress affects over 60% of the dry bean production worldwide (White and Singh, 1991). As described earlier, the bean growing areas in Latin America most affected by drought are Northeastern Brazil and the central and northern highlands of Mexico. These are the areas where drought tolerance screening has been undertaken. In Africa, droughts are frequent and severe in bean growing areas of eastern Kenya and eastern Transvaal, while other areas such as parts of northern Tanzania, the Kasese area of Uganda, and parts of the Hararghe Highlands and the Rift Valley of Ethiopia are affected by water deficits (Wortmann et al., 1998).

Breeding for drought resistance has a long history in Mexico, Honduras, and Brazil, and at CIAT in Colombia. It has gained momentum in recent years, with field studies of advanced lines in Cuba, Ecuador, El Salvador, Guatemala, and Nicaragua in Latin America, and with the creation of international drought nurseries in Kenya, Sudan, Ethiopia, and other countries in Eastern Africa. Most research has concentrated on germplasm evaluation. For example, White et al. (1994a,b) carried out genetic studies of parental materials from CIAT and from Mexico, finding that combining ability was determined largely by local adaptation. Thus, Mexican lines were good parents in Mexico and lines selected in Colombia served better as parents in Colombia, indicating that genes that were specific for drought resistance required an adapted genetic background for expression.

In the drought prone region of northern Mexico, two different sets of germplasm were tested under the typical conditions of insufficient and erratic rainfall. The primary trait measured was seed yield. The first set included more than 7000 accessions from the INIFAP (Instituto Nacional de Investigaciones Forestales, Agrícolas y Pecuarias) germplasm bank grown in subdivided sets over 3 years at two locations. Drought-resistant genotypes were identified mainly in the Durango (type III growth habit) and Mesoamerica races (types II and III), whereas genotypes from the Jalisco race were susceptible. The second set included 800 bush genotypes of the worldwide core collection assembled at CIAT (Tohme et al., 1995). After 2 years of evaluation, a set of 20 genotypes mostly from the Durango race originating in Mexico were identified as drought-resistant, plus a few accessions from other latitudes.

Subsequently, all resistant genotypes, along with locally improved cultivars previously identified as drought-resistant, were tested in eight trials across three different regions in Mexico: lowland, mid-altitude and highlands. In the four trials at the lowland tropics and in the mid-altitude El Bajio region, “G” accessions from the CIAT core collection (mostly Pinto landraces from the Durango race) performed poorly, as expected. In the former location, this was mainly due to a short growing cycle (due to photoperiod sensitivity coupled with short winter days), whereas in the latter location it was due to an inherent susceptibility to diseases (rust and common blight) and to leafhoppers (*Empoasca kraemeri*). In the lowland and mid-altitude sites, locally adapted genotypes along with introduced bred cultivars were among the top 25% yielders, a few of them yielding well in the four trials, e.g., TLP 19 and SEA 10, improved CIAT lines from the Mesoamerican race, and 97-RS-101 from the Durango race. In contrast, at the semi-arid highland location, CIAT “G” accessions were outstanding under both rainfed and rainfed-plus-irrigated conditions. In the four semi-arid highland trials, superior cultivars included the improved cultivar Pinto Villa and landraces G 13637 (Apetito) and G 842 (PI 201331). Furthermore, cultivars 97-RS-101 and SEA 10 were among the top yielders in six out of eight trials, and Pinto Zapata and 97-RS-110 in five trials. Superior cultivars at each site included genotypes from the type II and type III growth habit, early to mid-season types and those with disease resistance (Acosta-Gallegos et al., 2004). In addition, cultivars that displayed broad adaptation across regions were of neutral reaction to photoperiod. Under both terminal and intermittent drought stress, an accelerated partitioning of photosynthates toward the reproductive structures under stress seemed to be the chief trait for seed yield (Rao, 2001; Rosales-Serna et al., 2004; Rao et al., 2006a).

Singh (1995) reported the results of breeding for drought resistance in a tropical environment at CIAT in Colombia. One line, SEA 5, was especially resistant (Singh et al., 2001; Terán and Singh, 2002b). At higher latitudes in Mexico, cultivars Pinto Villa, and Pinto Saltillo were developed in and released for drought stressed environments of the northern highlands (Acosta-Gallegos et al., 1995; Sánchez-Valdez et al., 2004). They are extensively used in commercial production and as parents in the bean breeding programme. Although successful cultivars have been released (Beaver et al., 2003), sources of resistance outside the Durango race are being sought, based on the hypothesis that different mechanisms to cope with drought stress might exist in other genepools, plus the need to widen the genetic base in the Mexican breeding programme. A few promising cultivars from the Middle American (SEQ 12, SER 16, Negro Cotaxtla 91, Negro Veracruz, Black Jack) and Nueva Granada (ICA Palmar, A195) races have been identified. Muñoz-Perea et al. (2006), working in Idaho, found that Durango landraces of the red Mexican grain class presented a relatively better yield under drought than bred cultivars released over the past 30 years, whereas improved varieties released in other grain classes were superior to landraces. In other recent efforts, Beebe et al. (2008) have reported improvement of lines of small-seeded race Mesoamerica commercial classes (small red, small black and cream striped or carioca types). Besides improved yield under drought, the drought-selected lines presented shorter days to maturity, improved yield per day and, in some cases, better yield potential under favorable conditions.

The perspective of genepool structure, and especially of races, offers useful insights into past successes in breeding for drought resistance. Singh et al. (1991) noted that the race Durango often presents drought resistance and a good harvest index (HI). Much of the subsequent work on drought resistance has been built around the race Durango or genes extracted from it. The combination of Durango and Mesoamerica races has resulted in lines with higher yield in drought environments (Singh et al., 2001; Terán and Singh, 2002a) as well as in non-stressed environments (Nienhuis and Singh, 1986). This combination of races has also resulted in improved materials in other reports (Schneider et al., 1997a; Frahm et al., 2004; Beebe et al., 2008). Thus, these two bean races have complementary genes and/or mechanisms that permit the expression of transgressive segregation for drought resistance. It is notable that more progress has been made in small-seeded Mesoamerican types for Central America and Brazil, than in the large seeded Andean types that are more popular in Africa and parts of South America. This is possibly because crosses have rarely been made between Durango drought tolerance sources and Andean genotypes.

Schneider et al. (1997b) studied the genetics of drought resistance in Mexico and in Michigan, USA using quantitative trait loci (QTLs) detected with RAPD markers and multiple regression analysis. Four markers in one population and five in a second population of RILs were reported as important for drought resistance, although all of the non-anchored linkage groups were associated with some yield trait in some site and year. In a simulation of the application of MAS for a drought resistance QTL, the authors found that MAS would have been effective in one population in Mexico, and in the other population in Michigan. A second experience in the identification of markers for QTLs was reported by Beebe et al. (2006b). A population of RILs of the cross of SEA 5 × MD 23-24 was evaluated under drought and irrigated conditions in two seasons with contrasting patterns of drought. One QTL was common to two drought seasons, one QTL was specific to each of two seasons, and some were common to unstressed environments. What was perhaps most significant was that in no case were the two alleles at an important locus specifically adapted to the contrary environments (i.e., one allele to drought conditions and the other allele to favorable conditions). Rather, a drought allele (or an allele for a favorable environment) was accompanied by a neutral allele for the other environment. This implies that yield under drought and yield under well-watered conditions are not mutually exclusive and can be combined. In fact, cultivars that are high-yielding under irrigated conditions have shown, despite a large reduction, higher than average yields under terminal drought-stressed conditions (Acosta-Díaz et al., 2004).

### Physiological mechanisms of adaptation to drought

Adaptation to drought encompasses a diversity of mechanisms that enable plants to survive and produce in periods of dry weather. The mechanisms of drought resistance are grouped into three categories: drought escape; drought avoidance; and drought tolerance (Levitt, 1972).

Drought escape is defined as the ability of the crop to complete its life cycle before serious soil and crop water deficits develop. This mechanism involves rapid phenological development (early flowering and early maturity), developmental plasticity (variation in duration of growth period depending on the extent of water deficit), and remobilization of photosynthates to the grain.

Drought avoidance is defined as the ability of the crop to maintain relatively high tissue water potential, despite a shortage of soil moisture. It is achieved through increased rooting depth, an efficient root system and increased hydraulic conductance, and by reduction of water loss through reduced leaf conductance, reduced absorption of radiation by leaf movement/rolling, and reduced evaporation surface (leaf area).

Drought tolerance is defined as the ability of the crop to withstand water deficit with low tissue water potential. It is achieved through maintenance of turgor through osmotic adjustment (a process which induces solute accumulation in the cell), increase in cell elasticity and decrease in cell size, and desiccation tolerance by protoplasmic resistance. Blum (2005) indicated that an effective drought tolerance mechanism in crop plants is stem reserve utilization for grain filling under drought stress. Research approaches that have most successfully improved drought performance of crop plants: (1) used realistic soil conditions; (2) tested with adequate water and with limited water; (3) understood the sources of crop failure in the proposed growing area; and (4) targeted a limited number of traits for genetic improvement (Boyer, 1996).

Significant research efforts have been made, particularly over the past two to three decades, to improve common bean adaptation to drought (Laing et al., 1984; White and Singh, 1991; Subbarao et al., 1995; Rao, 2001; Amede et al., 2004; Hall, 2004; Ishitani et al., 2004; Beebe et al., 2008; Beebe, 2012). These have involved:

- Studying the effects of drought stress on plant growth, development, and seed yield (Robins and Domingo, 1956; Acosta-Gallegos and Kohashi-Shibata, 1989; White and Izquierdo, 1991; Nielsen and Nelson, 1998; Nleya et al., 2001; Ontiveros-Cortes et al., 2005).
- Developing field screening methods (Bascur et al., 1985; Sponchiado et al., 1989; White and Castillo, 1989).
- Evaluating and identifying sources of drought tolerance in germplasm (Da Silveira et al., 1981; Miller and Burke, 1983; Jara-R, 1990; White and Singh, 1991; Singh, 1995; Terán and Singh, 2002a,b; Muñoz-Perea et al., 2006; Singh, 2007).
- Evaluating physiological traits related to underlying mechanisms of adaptation to drought (see Table 1 for details and references).

**Table 1:** **Physiological studies with a focus on shoot and/or root traits that contribute to improved adaptation to drought in common beans**.

**Studied**	**Shoot traits measured**	**Root traits measured**	**References**
**NUMBER OF GENOTYPES**			
1	Photosynthesis, transpiration, leaf water potential, ribulose bisphosphate carboxylase (Rubisco) activity	–	O'Toole et al., 1977
3	Leaf water potential, osmotic potential, turgor potential, relative water content	–	Parsons and Howe, 1984
2	Leaf area, leaf dry weight, stem dry weight, leaf water potential, leaf osmotic potential, stomatal resistance	Rooting depth, root dry weight, root distribution	Markhart, 1985
4	Seed yield, crop dry weight, leaf area duration, number of seeds/pod, canopy temperature	Root length density	Sponchiado et al., 1989
6	Seed yield	–	White and Castillo, 1989; White et al., 1990
27	Carbon isotope discrimination, ratio of intercellular to ambient CO_2_ concentration, transpiration efficiency	–	Ehleringer et al., 1991
2	Water potential, relative water content, photosynthesis, chlorophyll fluorescence, quantum yield	–	Castonguay and Markhart, 1991
16	Seed yield, shoot dry weight, harvest index (HI), leaf conductance, days to maturity	–	White and Castillo, 1992
12	Net photosynthesis, transpiration, leaf nitrogen, specific leaf area, leaf conductance, photosynthetic nitrogen use efficiency, carbon isotope discrimination	–	Comstock and Ehleringer, 1993; Kao et al., 1994
9-parent diallel	Carbon isotope discrimination, leaf optical density, leaf N and K, relative duration of podfilling, shoot dry weight, HI, seed yield, 100 seed weight	–	White et al., 1994a
20	Days to flowering, days to maturity, phonological plasticity, relative response to photoperiod	–	Acosta-Gallegos and White, 1995; Foster et al., 1995
3	–	Root length density, root efficiency in water absorption	Guimarães et al., 1996
5	Stomatal conductance, water content, relative water content, moisture retention capacity, HI, phenology, biomass and yield components	–	Pimentel et al., 1999; Ramírez-Vallejo and Kelly, 1998; Serraj and Sinclair, 1998
4	Relative growth rate, leaf water potential, stomatal conductance, net assimilation rate, osmotic adjustment	–	Costa Franca et al., 2000
4	Leaf area index (LAI), shoot biomass, number of pods per plant, grain yield	–	Dowkiw et al., 2000; Gomes et al., 2000
2	Water potential, stomatal conductance, assimilation rate, leaf abscisic acid (ABA)	–	Menuccini et al., 2000
4	Leaf area, relative water content, transpiration rate, stomatal conductance, leaf dry mass, stem dry mass, shoot dry mass, seed yield	Root dry mass, root distribution	Mohamed et al., 2002
1	Leaf water potential, leaf osmotic potential, leaf turgor potential, sugars and sugar alcohols, proline	–	Amede and Schubert, 2003a
1	Leaf water potential, plant dry weight, stomatal conductance, water-use efficiency (WUE), leaf sugars	Root dry weight	Abebe and Brick, 2003; Amede and Schubert, 2003b
2 RIL	Seed yield, yield components populations	–	Frahm et al., 2004; Pastenes et al., 2004, 2005
2	Relative water content, shoot water content, photosynthesis, transpiration, WUE, leaf area, shoot dry mass, 100 seed weight, seed set, seeds/pod, pod length, number of pods, seed yield	Root dry mass, root distribution, root depth	Mohamed et al., 2005; Wakrim et al., 2005
6	Seed yield, HI, leaf area, relative growth rate, relative water content, leaf water potential, leaf osmotic potential, leaf turgor pressure, photosynthesis, leaf ABA, stomatal conductance, dark respiration, leaf sugars, leaf starch, stem sucrose, seed starch, seed protein	–	Gebeyehu, 2006
24 (2 for detailed analysis)	Seed yield and yield components, abscission of reproductive organs, relative growth rate, relative water content, stomatal conductance, transpiration rate, photosynthetic capacity, leaf ABA, leaf rotation, chlorophyll fluorescence, leaf anthocyanin and malondialdehyde	–	Lizana et al., 2006
2	Photosynthetic rate, chlorophyll fluorescence, electron transport rate, leaf area, leaf thickness, carotenoid composition, stomatal density, leaf cell organization	–	Wentworth et al., 2006
16	Biomass yield, seed yield, HI, 100 seed weight, days to maturity	–	Muñoz-Perea et al., 2007
2	Relative growth rate, photosynthesis and transpiration rates, stomatal conductance, water-use efficiency, relative water content, proline accumulation, glycolate oxidase activity, peroxidation, antioxidant enzyme activities, ascorbate, phenolic and flavanoid compounds	–	Rosales et al., 2012

Common beans are grown over a wide range of habitats where they can be exposed to seasonal droughts and wide fluctuations in soil moisture availability between years. Therefore, they have evolved several mechanisms to maintain plant water status within reasonable limits for normal metabolic functioning under drought stress (Beebe, 2012). Results from a set of the same genotypes that were evaluated in several countries in the 1980's indicate that local adaptation is an important component of drought resistance (White, 1988). A number of shoot and root traits contribute to improved drought adaptation. The root traits maximize water uptake, and the shoot traits optimize the use of absorbed water for producing grain during drought stress. Loss of leaf area is the most important morphological adaptation. It results from a reduced number of leaves, reduced size of younger leaves, inhibited expansion of developing foliage, or leaf loss accentuated by senescence, all of which result in decreased seed yield (Acosta-Gallegos, 1988). Through field screening, some relatively drought-tolerant lines of bean germplasm have been identified, such as BAT 477, A 195, and BAT 1289 (White, 1988; White and Singh, 1991). The superior adaptation of BAT 477 to water deficits was attributed to drought avoidance through greater root length density and deeper soil moisture extraction (Sponchiado et al., 1989).

White and Castillo (1992) grafted diverse shoot genotypes onto selected root genotypes of common beans and evaluated yield under drought. They found variation with shoot genotype, but the effect on growth and yield under drought was found to be small, compared with the effect of root genotype. Sanders and Markhart (1992) also used grafting to examine the importance and mechanisms of the root system's effect on leaf water status in *P. vulgaris* and *P. acutifolius*. They found that the root genotype determined leaf water potential in the most stressed plants, and that roots of tepary beans had greater hydraulic conductivity than those of common beans. Castonguay and Markhart (1991) measured saturated rates of photosynthesis in water-stressed leaves of common and tepary beans, and found that genotypic variability in drought tolerance between the two was not related to differences in mesophyll tolerance of dehydration. Tepary beans relied more on drought avoidance than on drought tolerance. Severe drought impaired nitrogen mobilization, HI and water-use efficiency (WUE) in common beans (Foster et al., 1995).

Further research work by White (1993) under field conditions indicated that WUE (based on carbon isotope discrimination, CID) was not a promising indicator of adaptation to drought. Since this work included a limited number of parental genotypes in a single year, further research work is needed on WUE using CID values in leaves and grain. Other physiological traits such as shoot dry weight and leaf nitrogen concentration appeared the most promising based on heritability, strong general combining ability effects, and correlations with seed yield across trials (White et al., 1994a,b). Phenotypic plasticity is considered to be another mechanism contributing to increased performance under drought (Acosta-Gallegos and White, 1995). This particular attribute, accentuated in photoperiod-sensitive cultivars, allows genotypes to shorten their growing cycle dramatically at later planting dates to avoid drought conditions later in the growing season.

Rao et al. (2004) evaluated 36 promising bred lines and accessions under field conditions over two seasons at CIAT in Colombia, and found that two accessions of *P. acutifolius* (G 40159 and G 40068) and two bred lines (RAB 650 and SEA 23) were outstanding in their adaptation to water stress conditions. The superior performance of these two accessions under drought was associated with their ability to mobilize photosynthates to the developing grain and to utilize the acquired nitrogen more efficiently for grain production. More recent field evaluation of advanced lines at CIAT resulted in identification of three lines (SER 16, SEA 5, and SER 5) that were superior in their adaptation to drought stress conditions (Rao et al., 2006a). The superior performance of these lines was associated with higher values of pod harvest index (PHI), pod partitioning index and leaf area index (LAI), and a lower proportion of pod wall biomass and lower value of seed phosphorus content. The findings indicate the importance of greater mobilization of photosynthates to pods and seed per unit of seed phosphorus in common beans under rainfed conditions. The SER lines that were developed in the last few years seem to combine these desirable traits for drought adaptation (Beebe et al., 2008). The above field studies conducted at CIAT have contributed to the analysis of phenotypic differences in shoot traits that contribute to superior adaptation to drought stress conditions. From these studies, it has been learned that superior PHI, pod partitioning index and lower proportion of pod wall biomass are important phenotypic traits that reflect greater ability to mobilize photosynthates to grain under drought stress. Recently, Klaedtke et al. (2012) reported that photosynthate mobilization capacity from drought adapted common bean lines can improve yield potential of interspecific populations within the secondary gene pool.

The candidate genes underlying drought tolerance are beginning to be understood at a molecular level as well as for their physiological effects (Ishitani et al., 2004). At CIAT, candidate genes for drought tolerance are being pursued based on genes for osmotic adjustment, transpiration/WUE, and root development. Some, such as the dehydration responsive element binding protein (DREB) genes, could be converted to molecular markers for physical mapping and MAS. Subtractive libraries based on differential display of genes expressed in roots under stressed and non-stressed conditions, or by tolerant and sensitive genotypes, may lead to the identification of genes specific to the deep-rooting trait.

## Methodologies for improving adaptation to drought

### Breeding strategies

Since the initiation of the breeding effort in the semi-arid highlands of Mexico, local landraces from the Durango race have been utilized in the development of improved cultivars, along with sources of specific traits, mostly disease resistance and earliness. Successful cultivars include in their pedigree parents from the Nueva Granada race chosen on the basis of yield, disease resistance and earliness (Acosta-Gallegos et al., 1995; Beaver et al., 2003; Sánchez-Valdez et al., 2004). The yield testing of the bush core collection in the semi-arid highlands of Mexico mostly identified accessions from the Durango race as resistant under intermittent drought stress. The resistant accessions are of indeterminate prostrate type III growth habit, root-rot resistant, photoperiod sensitive and originating in the region (Acosta-Gallegos et al., 2004). Under intermittent stress environments, mid-season genotypes of indeterminate prostrate growth habit that flower in flushes are best suited to cope with such variable conditions (Acosta-Gallegos and Kohashi-Shibata, 1989; Rosales-Serna et al., 2004).

For the improvement of small seeded cultivars of the race Mesoamerica, Durango genes continue to be valuable, but these are now introgressed into this race, so no direct use of Durango is being practiced. For Central America, Northeastern Brazil, and the Caribbean, lines in the small red, small black, and carioca grains are being developed, which present double or more yield under severe stress compared to the respective commercial control (Beebe et al., 2008). Small seeded beans are often planted in warmer climates where high temperatures exacerbate drought, or under conditions of low soil fertility or aluminum toxicity that can limit vigor and root development. Thus, tolerance of low soil fertility, especially to low soil phosphorus availability, and heat tolerance should be combined with drought resistance. The combination of drought and low soil fertility tolerance has proven to be practical, since several drought-resistant lines already express a relative degree of tolerance to low soil phosphorus availability (Beebe et al., 2008). If progeny of interspecific crosses with *P. acutifolius* become available as sources, they will probably be small seeded, and thus of more immediate use to improve small seeded cultivars. Similarly, to date, it has been easier to introgress genes for aluminum resistance from *P. coccineus* into the small seeded genotypes than into the large seeded Andean types. These are all options to be pursued. Furthermore, in the light of results that demonstrated that differences in photosynthate mobilization during terminal drought were related to drought resistance, field selection for well-filled grain has been used to improve drought resistance in the small red and black seed classes (Beebe et al., 2008). This trait apparently integrates the effects of several elements of a physiologically complex process, and the practice of selecting for good seed filling has worked well when terminal drought was severe enough to have visible effects on seed quality.

Within the Andean genepool, drought resistance is needed most in bush beans, since climbing beans are usually planted in moister environments. Parental sources combining the race Durango with Andean types have resulted in lines with modest gain over the Colombian cultivar ICA Quimbaya—one of the best Andean genotypes available previously—and have produced resistant lines in a much wider range of grain colors (CIAT, 2006). Screening of potential parental genotypes has identified drought resistance in the SEQ and BRB series of advanced lines from CIAT, as well as in some dark red kidney (DRK) genotypes derived from ICA Quimbaya. In the case of Andean beans, drought resistance traits often need to be combined with vegetative and reproductive heat tolerances, especially for intermediate elevation production sites since, with the exception of some heat-tolerant germplasm accessions, Andean beans are notably poor at seed set in high temperatures.

The breeding strategies needed for improvement of drought tolerance in commercial classes of either genepool must take into account the quantitative nature of inheritance of this trait. This fact will circumscribe the breeding methods that can be applied to drought resistance, and calls for the application of novel approaches that are not widely practiced in common bean breeding. A particularly useful method for drought resistance breeding where sufficient drought resistance is found within a given genepool is recurrent selection (Beebe et al., 2008). Prebreeding can be used to create a sufficient number of potential parents with drought resistance component traits to initiate recurrent selection. After generating drought-resistant advanced lines, these can be used with standard common bean breeding techniques to incorporate new traits into the drought-resistant background. Among these techniques, gamete selection is a method recommended for use in common beans. It involves complex crosses and selection among F_1_ plants and F_1_-derived families (Singh, 1994), whereas pedigree selection is widely practiced by bean breeders to obtain fixed lines (Miklas et al., 2006).

Another method termed “advanced backcrossing” is a potentially useful method for improving drought resistance traits using crosses across genepools. Advanced backcrossing is valuable because it can be used to transfer multiple gene combinations from source germplasm to recipient genotypes. One advantage of the advanced backcross technique is the creation of improved lines that are useful simultaneously in an agronomic context and for genetic analysis. Blair et al. (2006b) showed that advanced backcrossing could be used for both QTL detection and discovery of transgressive segregation for yield traits in common beans. In summary, it can be seen that, whether the approach is advanced backcross, recurrent, gamete, or pedigree selection, good drought resistance sources must be amply represented in the genetic makeup of any of the populations developed.

While resistance to local diseases is a requisite for cultivar development in general, resistance to soil pathogens is especially important in drought-prone areas. Soil pathogens can infect when moisture is adequate and inhibit the root growth that is necessary for drought resistance later in the season. *Fusarium* spp in Mexico exacerbate drought by causing deterioration of the root system and reducing absorptive capacity for nutrients and water (Navarette-Maya et al., 2002). Charcoal rot caused by *Macrophomina phaseoli* is most severe under drought (Frahm et al., 2004). If early rains are abundant and result in serious infection and damage to hypocotyls and tap roots by fungi such as *Rhizoctonia solani* or *Sclerotium rolfsii*, this will also expose the crop to more severe late season drought.

As yet, there has been no routine use of MAS for improving drought resistance in common bean breeding programmes. However, MAS offers great potential for incorporating disease resistance into drought-resistant genetic backgrounds. The application of MAS for resistance to bean golden yellow mosaic virus (BGYMV) is the best example to date of selection of QTLs in common beans (Miklas et al., 2006). Two important QTLs have been tagged, sequence characterized amplified region (SCAR) markers created, and protocols defined. Selection has been practiced on populations ranging from F_1_ plants of complex crosses to advanced families. Subsequently, it will be necessary to confirm resistance of lines in field trials because the full resistance complement cannot be assured by MAS, markers run the risk of genetic recombination and, finally, an entire suite of traits is required in a commercial cultivar. Application of MAS for drought would follow a similar scheme: the identification of relatively important QTLs and the creation of robust markers, followed by their use in various generations including in the F_1_ of complex crosses, and subsequent field evaluation to confirm drought resistance and to distinguish levels of resistance.

However, compared to MAS for disease resistance, QTLs for drought resistance would require an additional step to validate the value of QTLs over sites, seasons, patterns of drought, soil types, etc. The nature of drought and its interaction with multiple environmental factors make the validation of QTLs much more complex. Ideally, the validation would be carried out within the target production zone, but this is normally difficult, since finding uniform experimental conditions for evaluation of large RIL populations close to production zones is usually not practical. A compromise might be to test a subsample of 30–40 phenotypically extreme segregant RILs in a smaller trial over multiple sites, with the sole purpose of validating the QTL. This population size could serve to confirm the effect of a relatively major QTL, assuming that this is the target for eventual MAS. Schneider et al. (1997b) were able to validate markers using a small set of selected RILs. In this scheme, a multi-trait analysis (considering yield at different sites as independent traits) can augment the statistical power lost due to small population size (Jiang and Zeng, 1995).

### Trial planning

In planning trials for drought resistance testing it is important to consider carefully the choice of field sites and management of collateral factors. Principal among these are seasonal rainfall patterns, aspects of water control provided by irrigation systems and/or rainout shelters, soil bulk density, and prevalent abiotic and biotic stresses including soil fertility/toxicity problems and diseases or insect pests, some of which are more prevalent during dry season testing.

Crop yields in farmers' fields are as much affected by the timing of water deficits during a season as by the total seasonal water supply (Passioura, 2007). Field evaluation under realistic production conditions is the “gold standard” of drought resistance. In general, reliable and uniform field agronomy continues to be the key to genetic advance. This requires a uniform soil profile and texture, to the extent possible, since these affect the available soil moisture. Sampling of soil cores can reveal hidden soil variability that does not affect crop performance under optimal conditions but that would later affect the crop response under stress. Uniform field preparation with regard to subsoiling, plough depth, and bedding is critical, since this determines much of the effective rooting volume of the crop. Often, spatial variability in reaction to drought can be traced to variability in soil preparation. Gradients are often observed down the length of the field in the direction of field preparation. In this case, experimental designs might best be oriented in the same direction instead of across the field, and lattice designs often reduce experimental error. The testing of genotypes under stress and non-stress conditions allows for the gathering of more information in a single season and site, and for the calculation of several indices such as the geometric mean, the reduction of the yield, and the drought susceptibility index (Fischer and Maurer, 1978). As a rule of thumb, the more severe the drought-stressed environment, the more replicates are needed.

The interaction of drought with other stresses is notorious, especially with edaphic stresses (fertility, toxicities, and high soil bulk density) that affect root development. Also, drought effects are frequently exacerbated by pathogens causing root rots—*Fusarium* spp and *Rhizoctonia solani* in highland environments and *Macrophomina phaseolina* in lowland environments. If these are relevant stresses in the target production zones, it is important to understand their impact on the expression of drought resistance in order to have a realistic expectation of the benefits to be derived from drought resistance. For example, in Nicaragua, drought-resistant lines in fertile environments yield 50% more than local varieties under drought, but only 15% more in infertile environments (Llano, pers. communication). This requires a careful strategy that takes into consideration all factors and allows interactions among stresses to be examined. Inclusion of multiple stresses during the selection process is normally too complex and would obscure useful genetic variability in drought resistance. It is more practical to practiced selection for individual stresses in tandem, and to study the reaction to combined stresses with advanced lines.

Biotic stresses that are more prevalent during dry season testing include a range of insect pests such as leafhopper and whiteflies. These must be controlled for valid testing of drought resistance, since these pests can have a large effect on plant phenotype either through direct feeding or virus transmission. It is often necessary to control soil fungal pathogens, such as *Macrophomina* and *Sclerotium*, which are a major concern under water stressed conditions and which are easily spread through irrigation water used to establish drought nurseries. This can be achieved by seed dressing and directed fungicide application early in the growing season, or by planting into plots that have not had a long history of common bean testing. This is often the only solution when faced with high levels of *Fusarium* infestation.

Segregating population analysis in particular requires uniform conditions of soil and stress, and is best managed with the interplanting of parental genotypes plus frequent checks throughout the field for ready comparison. As noted above, variability in crop response often follows lengthwise field preparation. Thus, the identification of superior early generation materials for which replication is not practical must take into account not only neighboring plots to the right and left but also in the same row above and below the plot of interest. Segregating populations can also be replicated across sites to minimize the effects of selection at a single site, where the risk of out-of-season rainfall can interfere with drought selection pressure. Selection for traits of high heritability such as disease resistance in early segregating generations, and delaying the selection for yield under drought stress to intermediate generations, are common practices.

For family and line evaluation, lattice designs are a valuable tool, especially to control error that results from soil variability. At the family stage of testing that normally involves several hundred entries, unbordered two-row plots can be used for economy of space. Small lattices permit even smaller sub-plots. A six-by-six lattice is relatively easy to accommodate in the field, with compact sub-plots that are two plots by three plots. Where a larger number of families and lines are to be evaluated, common controls among trials permit comparison of the relative degree of drought resistance across materials in different trials. Lattice-design experiments are also used effectively for testing of recombinant inbred line populations in QTL studies. After the numbers of families or lines have been reduced, duplicated trials under two moisture regimes at two locations allow for the identification of highly responsive genotypes or for those that show the least genotype-by-environment interaction (GEI). In environments with a history of severe drought stress, trials need to be established with a higher number of replications than usual, due to large experimental errors.

### Water stress management and characterisation

With irrigated conditions, drought may be predictable in both timing and intensity but, under rainfed conditions, unpredictability is the rule. In bean growing areas in the tropics, terminal drought stress is more common than intermittent drought stress. In Latin America, terminal drought stress affects Central America and Northeastern Brazil, while intermittent drought stress is common in the semi-arid highlands of Mexico. In Africa, terminal drought is more common than intermittent drought under the short rains (late October to January) in the Eastern Highlands, while in the long rains (February to June) and in regions of Ethiopia, intermittent drought is common.

The tap root of the bean plant may reach a depth of 1–1.5 m. The lateral root system is extensive and is mainly concentrated in the first 0.3 m. At emergence, the rooting depth is about 0.07 m, at the start of flowering it is 0.3–0.4 m, and at maturity 1–1.5 m. Water uptake occurs mainly in the first 0.5–0.7 m of depth. In areas of intermittent drought stress, indeterminate plants with profuse branching above and below ground (i.e., roots) are better equipped to cope with drought spells of variable duration. Under these conditions, lateral and even adventitious roots are important to take up moisture during the scarce rain events. Under conditions when evapotranspiration is 5–6 mm day^−1^, 40–50% of the total available soil water can be depleted before water uptake is affected. When water levels are reduced beyond this point and drought effects begin to occur, water stress in the plant can be detected by eye, because the leaves turn dark bluish-green in color. When the crop is grown for grain production, seed yield will be seriously affected if the soil water depletion level during the grain filling period reaches 60–70% of the total available soil water. The water utilization efficiency for harvested yield or crop water productivity for dry beans containing about 10% moisture is 0.3–0.6 kg m^−1^.

The complexities of water deficit are apparent when one considers the effects of variation in climatic and edaphic conditions on the extent of dehydration which develops in a crop, and possible interference from biotic stresses as cited above. Management aspects that improve drought adaptation include improvement of soil water holding capacity through incorporation of organic matter, reduction of soil erosion or improvement of tillage practices, development of water catchment systems, use of tied ridges, and changes in planting dates. These practices involve many location-specific considerations and require a cropping systems approach to production under water-limited conditions.

Achieving the desired level of stress is one of the most important and yet difficult facets of managing drought trials. Extreme level of drought stress could reduce seed yields to very low levels such that genotypic differences disappear, whereas insufficient stress could result in selection of non-resistant genotypes. Since very few bean growing areas in the developing world are dependent on irrigation, most strategies to manage drought stress have to focus on alternatives under rainfed conditions, with the possibility of supplemental irrigation. Trials could be established with the minimum amount of water needed to assure vigorous seedling establishment, and then irrigation is withheld to simulate terminal drought stress. Use of rainout shelters where available, can assure good terminal stress conditions. Use of furrow irrigation or sprinkler irrigation and withholding water at different growth stages of the crop can help to quantify the effects of water stress on crop growth, development, and yield.

Line source sprinklers offer a specialized irrigation system for producing a gradient of water stress or a range of levels of stress in the field. By closely spacing sprinklers along a single line, and planting genotypes in strips perpendicular to the line, water could be applied in a gradient. The main advantage of this system is to quantify the reaction of a genotype to different levels of stress, although wind speed and direction can influence the water stress gradient in the field.

### Water strategy

The status of water in soils, plants and the atmosphere is commonly described in terms of water potential (Ψ_w_) i.e., the chemical potential of water in a specified part of the system compared with the chemical potential of pure water at the same temperature and atmospheric pressure. It is measured in units of pressure (MPa; megapascal). The total water potential at any point in the plant can be partitioned into: (1) the osmotic potential arising from the presence of dissolved solutes; (2) the turgor potential arising from the forces exerted on the cell walls from the water attracted to the cell by the solutes and the solids in the protoplast; (3) the matrix potential arising from capillary or electrostatic forces associated with cell walls and colloidal surfaces; and (4) the gravitational potential arising from gravitational forces on the water in the plant.

Plants require vast quantities of water. Whereas they incorporate more than 90% of the absorbed nitrogen, phosphorus, and potassium, and about 10–70% of photosynthetically fixed carbon into new tissues (depending on respiratory demands for carbon), less than 1% of the water absorbed by plants is retained in biomass. The remainder is lost by transpiration, involving the absorption of water by the plant roots, the transport of water through the conducting tissues of the plant, and the passage of evaporated water through the leaves and into the air, primarily through the stomata. The essential need for water for crop growth, development and yield arises from four features of plants (Bennett, 2003): (1) When plants open the stomata of their leaves to admit atmospheric CO_2_ for photosynthesis, they lose water vapor through the same pores, a process known as “stomatal transpiration.” Stomatal conductance is more strongly correlated with several photosynthetic parameters (electron transport rate, carboxylation efficiency, intrinsic WUE, and respiration rate in the light) than with leaf water status (Medrano et al., 2002). (2) Leaves and stems may lose water by transpiration through non-stomatal surfaces even when stomata are closed. (3) Transpiration serves to cool leaves that are exposed to high air temperatures, low atmospheric water vapor pressures, or the heating effect of light (Radin et al., 1994). (4) The transpiration stream also serves to transport to the leaves both inorganic nutrients from the soil and a range of chemicals synthesized in the roots, including signal molecules that contribute to the integrated response of the whole plant (Peuke et al., 2002). Thus, the growing bean crop will transpire several hundred times more water than is present in its tissues at any one time.

Bean cultivars adapted to drought would require less water for irrigation and would, therefore, contribute to the conservation of an important natural resource. The short growing season reduces water requirements in common beans to levels below those of other species generally considered as more drought-adapted (White, 1993). The water requirements of a bean crop depend on its environment and nutrition. Water infiltrates the pores between soil particles and is held there with varying degrees of tenacity. Water tension (a negative pressure) in soil at any moment controls the movement of soil water in the soil and its use by plants. This water tension is expressed in units of MPa. When tension is low (between −0.01 and −0.03 MPa), water moves to lower soil layers because of gravitational pull. But when soil water tension is −1.5 MPa or less, the adhesive force is so strong that plant roots can hardly extract water from soil. At approximately this water tension, most crops permanently wilt and stop growing. The permanent wilting point is species specific and in the case of common bean, soil water tension values above at or lower than −0.8 MPa could impose significant drought stress and limit grain yield. Soil water at a tension between about −0.01 and −1.5 MPa is considered available for plants.

An assorted range of methods and instruments have been developed to measure and express soil water. Basically there are three ways: (1) weight percentage; (2) volume percentage; and (3) tension. The choice of whether to express soil water content on a weight or a volume basis is not a critical one if the information necessary to convert one to the other is also provided. Field capacity (FC) is defined as the water content after the soil becomes saturated, followed by complete gravitational drainage. There is a higher soil water content at FC in fine-textured soils with a high clay or organic matter content. The amount of available water is higher in clay than it is in sandy soils. If the bean crop does not receive enough water either through rainfall or through irrigation to maintain leaf expansion and high rates of net photosynthesis per unit leaf area, total canopy dry matter accumulation will decline, crop development will be affected, and grain yield will be reduced. The extent of yield loss is very much dependent on the timing, duration and intensity of water deficit.

Water requirements for maximum production of a 60–120 day bean crop vary between 300 and 500 mm depending on climate (Allen et al., 1998). Crop coefficient (K_c_) values that relate reference evapotranspiration (ET_o_) to water requirements (ET_m_) for different development stages of dry beans are: during the initial stage 0.3–0.4 (15–20 days), during the development stage 0.7–0.8 (15–20 days), during the mid-season stage 1.05–1.2 (35–45 days), during the late-season stage 0.65–0.75 (20–25 days), and at harvest 0.25–0.3.

Guerra et al. (2000) found the highest bean seed yield with irrigation at -41 kPa soil water tension measured at a soil depth of 10 cm. Recently, Muñoz-Perea et al. (2007) examined differences among dry bean landraces and cultivars (pinto and red market classes) in terms of WUE under intermittent drought-stress and non-stress environments. Under severe drought stress, WUE in pinto beans ranged from 1.5 to 4.4 kg ha^−1^ mm^−1^ water. Under favorable milder climatic conditions, the mean WUE value was 10 kg ha^−1^ mm^−1^ water in the drought stress environment and 8.7 kg ha^−1^ mm^−1^ water in the non-stress environment. Using one of the drought adapted small seeded red lines (SER 16), Builes et al. (2011) reported WUE values up to 9.2 kg ha^−1^ mm^−1^ water under drought stress.

Under rainfed conditions, water deficit can occur more than once during a crop's growth cycle, caused by erratic patterns of rainfall distribution, and may kill the crop under a severe and prolonged period of drought (Thung and Rao, 1999). The intensity and duration of stress determine the degree of yield reduction relative to yield potential. Different problems are created by water deficits at different key developmental stages of the bean crop, i.e., at sowing, establishment, branching, flowering, and grain filling.

All other factors being equal, genotypes with high WUE will survive and grow better in water-limiting environments than genotypes with low WUE. However, in nature, all other factors are rarely equal. The physiological basis for variation in drought resistance in common beans may be due to a wide and potentially unrelated array of mechanisms including earliness, rooting depth and distribution, carbon allocation patterns, leaf morphology, gas exchange patterns, osmotic adjustment, and photosynthate mobilization to grain. In general, selection for improved WUE through analysis of carbon isotopes will be most useful in selection for maintenance of growth under drought rather than survival. Survival mechanisms may relate more to growth phenology and carbon allocation patterns than improved carbon gain per unit water loss. Thus, increased survival under imposed drought could be related more strongly to allocation to roots than to gas exchange characteristics. It is possible that the lack of a positive relationship observed in common beans between carbon isotope discrimination (Δ^13^C) and seed yield under acid soil conditions, where root growth is restricted under dry conditions (White, 1993), may be due to genotypic differences in plant survival mechanisms.

### Phenotyping traits

The phenotype is a complex expression of the genotype and its interaction with the environment. Field trials for drought breeding and associated goals are normally conducted in the dry season of the year to determine genotypic differences for resistance. The trials could include germplasm accessions, advanced generation bred lines, and recombinant inbred lines as entries. Two levels of water supply (irrigated for no stress and rainfed for drought stress) need to be applied to quantify the effects of the intensity and duration of drought on crop growth and seed yield of genetically fixed materials. When the bean crop is grown with a sufficient water supply, the timing of irrigation is important and applications of water should be directed toward meeting water requirements during the establishment period, the early part of the flowering period, and at grain filling. Non-irrigated treatments generally receive water only during the establishment period, usually through pre-seeding and/or post-emergence applications of water. Depending on the number of genotypes, a partially balanced lattice design (4 × 4 or 6 × 6 or 10 × 10) with three replications can be used. The field trials can be planted in continuous rows with each genotype per replication planted in four rows of 5 m length with a row-to-row distance of 0.6 m and a plant-to-plant spacing of 0.075 m (with 15 seeds for a 1 m-long row to have a final number of 10–15 plants per 1 m-long row). The middle 2 rows are used for seed yield determination.

Climate data (daily rainfall, minimum and maximum temperature, relative humidity, and pan evaporation) need to be recorded. Depending on the rainfall and soil texture, two to three gravity irrigations are needed to establish the trials with control and drought treatments (one irrigation at 6 days before planting, and another irrigation at 10–12 days after emergence). The specific management practices and amount of water to be applied by either furrow or sprinkler irrigation will need to be calibrated empirically for local conditions. However, the rule of thumb should be to seek a 60–80% yield reduction in susceptible controls compared to the irrigated treatment, assuming yield potential of around 2.5 t ha^−1^ in control plots. In other words, the susceptible controls would ideally yield in the range of 0.5–1 t ha^−1^ to have maximal discrimination among genotypes and better chances of selecting true drought resistance. The control treatment will require additional irrigations (four to five) depending on the rainfall. The drought treatment will not receive any additional irrigation if furrow irrigation is used, but to induce drought stress with sprinkler irrigation, a reduction of about 50% in the amount of water applied to control plots may be considered. It is important to monitor the amount of water applied (e.g., 35–50 mm) for each furrow irrigation. Also, soil samples from each replication (that includes all genotypes) need to be collected at the time when irrigation is stopped for rainfed treatment, and followed at flowering, mid-podfilling and at physiological maturity. Soil samples need to be collected with a soil corer up to 80 cm in depth (at 0–5, 5–10, 10–20, 20–40, 40–60, 60–80, and 80–100 cm) to quantify soil moisture content gravimetrically. This includes weighing the fresh and dry weight of each soil sample for each soil depth. These measurements will allow quantification of the degree of drought stress at different growth stages.

Crop development needs to be monitored by recording days to flowering and days to maturity. For quantifying physiological differences in drought resistance, a number of plant attributes can be measured at the mid-podfilling growth stage. To measure plant attributes, a row length of 0.5 m (0.3 m^2^ area) for each plot should be selected for destructive sampling. During the sampling, the plants are counted (number per 0.5 m) and cut to the soil surface, put in a plastic bag and transported to the station or field room to process. Plants are separated into leaves, stems and the remaining plant parts (pods and reproductive structures). If a leaf area meter is available, the leaf area can be determined. The plant parts need to be put in separate paper bags for oven drying (70°C for 2 days). After drying of the samples, the dry weight of each is recorded. From these dry weights, total dry matter production and dry matter distribution into different plant parts as well as the leaf to stem ratio at mid-podfilling can be quantified.

Yield components should be measured at harvest time. Again, a 0.5 m long row (0.3 m^2^ area) is selected, and the number of plants counted and cut to the soil surface. The plants are put into a paper bag and transported to the station or field room. They are separated into stems and pods, and the number of pods and number of seeds per harvested area counted. The stem, pod wall and seed samples are oven dried at 70°C for 2 days and their dry weights recorded.

#### Target traits and how to measure them

Many drought adaptation traits, such as phenology, root size, and depth, hydraulic conductivity and storage of reserves, are associated with plant development and structure, and are constitutive rather than stress-induced (Chaves et al., 2003). Condon et al. (2004) have suggested that the consequences of various plant traits and environmental conditions have to be evaluated in the specific field environments in which the crop is to be grown. The target shoot and root traits that are pertinent for drought resistance breeding in common beans are described below.

#### Target shoot traits

From the phenotyping protocol described for field conditions, the following shoot traits that are related to seed yield can be quantified:

- At mid-podfilling: dry weights of leaf biomass, stem biomass, pods plus reproductive structure biomass, total shoot biomass, and leaf to stem ratio of dry weight.
- At harvest: dry weights of stem biomass, pod biomass and seed biomass, number of pods per plant, dry weight of pod wall biomass and proportion of pod wall biomass to pod biomass, seed number per pod, 100 seed dry weight, seed number per area and pod number per area.
- Seed yield: the two central rows of each plot are used to determine seed yield.
- Geometric mean (GM): this is determined for seed yield, 100 seed weight and days to maturity as GM = (ns × ds)^1/2^ where ns is no stress and ds is drought stress.
- Harvest index (HI): seed biomass dry weight at harvest/total shoot biomass dry weight at mid-podfilling × 100.
- Pod harvest index (PHI): the PHI for each genotype is determined by seed biomass dry weight at harvest/pod biomass dry weight at harvest × 100.
- Pod wall biomass proportion (%): pod wall biomass dry weight at harvest/pod biomass dry weight at harvest × 100.
- Pod partitioning index: pod biomass dry weight at harvest/total shoot biomass dry weight at mid-podfilling × 100.
- Stem biomass reduction (%): (stem biomass dry weight at mid-podfilling—stem biomass dry weight at harvest)/stem biomass dry weight at mid-podfilling × 100.
- Grain filling index (GFI): the GFI for each genotype can be estimated from 100 seed dry weight under rainfed conditions/100 seed dry weight under irrigated conditions × 100.
- Seed production efficiency (number g^−1^): seed number per area/total shoot biomass dry weight at mid-podfilling per area (adapted from Board and Maricherla, 2008).
- Pod production efficiency (number g^−1^): pod number per area/total shoot biomass dry weight at mid-podfilling per area (adapted from Board and Maricherla, 2008).
- Drought intensity index (DII): the DII for each growing season can be calculated as DII = 1 – *X*_ds_/*X*_ns_, where *X*_ds_ and *X*_ns_ are the mean of all genotypes under drought stress and no stress treatments, respectively.
- Drought susceptibility index (DSI) for seed yield: the DSI for each genotype is calculated as follows: DSI = (1 – *Y*_ds_/*Y*_ns_)/DII, where *Y*_ds_ and *Y*_ns_ are mean yields of a given genotype in drought stress and no stress environments, respectively (Fischer and Maurer, 1978).

#### Additional shoot traits

These include non-destructive measurements that are related to physiological processes such as photosynthetic efficiency, total chlorophyll content (Soil–Plant Analyses Development or SPAD measurement), stomatal conductance, transpiration rate, leaf temperature (in both the morning and afternoon), and leaf water potential. The destructive measurements that are related to growth and metabolism include LAI, canopy dry weight per plant (leaf, stem and pod biomass), shoot nutrient (nitrogen and phosphorus) uptake, shoot and seed ash content, and shoot and seed total non-structural carbohydrates (TNC). Seed nitrogen, phosphorus, ash content, and TNC can be measured at the time of harvest.

Field evaluation of 121 RILs of the cross MD 23-24 × SEA 5 over 2 seasons at CIAT in Colombia using the above phenotyping protocol resulted in identification of one line (MR 81) that was superior in its adaptation to drought stress conditions (Rao et al., 2005). The superior performance of this line was associated with higher values of PHI, pod partitioning index, HI, and seed TNC, and a lower proportion of pod wall biomass and lower value of seed phosphorus content, indicating the importance of greater mobilization of photosynthates to pods and seeds per unit of seed phosphorus in common beans under rainfed conditions.

#### Target root traits in the field

Root traits associated with drought tolerance can be measured either in the field or in the greenhouse, and these include root depth and root architectural traits. Rooting depth and root distribution under field conditions can be quantified using soil cores taken at different soil depths followed by root washing, scanning and weighing as described below for greenhouse root phenotyping. Deep rooting has been positively correlated with seed yield, crop growth, cooler canopy temperature, and soil water extraction in common beans (Sponchiado et al., 1989). In another study by White and Castillo (1988), drought-tolerant bean genotypes were able to extend their roots to a depth of 1.2 m in drought environments, whereas sensitive genotypes could not extend their roots beyond 0.8 m. These differences in rooting depth were reflected in overall shoot growth and seed yield.

#### Rooting behavior and shoot development under greenhouse conditions

When grown in the greenhouse, beans are planted in a mix of a soil (4–8% soil organic matter) with river sand (2:1 w/w) and grown for ca 35–45 days in small plastic tubes (80 cm long and 7.5 cm in diameter) covered with polyvinyl chloride (PVC) tubes. The plastic tubes are filled to 75 cm of their total length with 2400 g of moistened soil–sand mix (made by mixing a ratio of 500 g of soil to 100 ml of water and packing into the tubes in aliquots to ensure uniform settling). Trials are planted as a randomized block in a split plot arrangement with three levels of water supply: 80% FC (well-watered), 40% FC (simulation of intermittent drought), and without irrigation (simulation of terminal drought conditions) as main plots, and genotypes as subplots. Watering the plastic tube and allowing it to drain and then registering the amount of soil moisture left determines FC. Soil is fertilized with an adequate level of nutrients based on soil analysis. Water stress treatments can be imposed after 10–14 days of initial growth of the plants. The initial soil moisture level for the three treatments is 80% of FC. Plants in the well-watered (80% FC) and intermittent drought (40% FC) treatments are maintained by weighing each plastic tube every 3 days and applying water to the soil at the top of the plastic tube. Plants with terminal drought receive no water application after the initial establishment. Each plastic tube is weighed to determine the soil moisture content at 3-day intervals until harvest.

#### Traits measured in greenhouse trials

A number of shoot physiological characteristics are measured in a soil tube screening system assay. These include photosynthetic efficiency, total chlorophyll content (SPAD), stomatal conductance and transpiration rate, leaf temperature (both in the morning and afternoon), and leaf water potential. At the time of harvest (ca 35–45 days after planting and 3 weeks of drought stress), leaf area, shoot biomass distribution (leaf, stem, pod, and root biomass), leaf TNC content, and root characteristics are determined. The soil tube is sliced into 5 layers (0–5, 5–10, 10–20, 20–40, and 40–75 cm). Roots in each soil layer are washed free of soil, and length, diameter, specific root length, and dry weight are determined. Root length and diameter are measured with an image analysis system (WinRHIZO, Regent Instruments Inc.)^1^. Root weight is determined after the roots are dried in an oven at 60°C for 48 h.

Rao et al. (2006b) used the above soil tube screening system to evaluate the impact of drought on different genotypes of common beans in terms of root growth and root distribution. Results on five genotypes grown in large soil cylinders indicated that SEA 5, BAT 477, and G 21212 were deep rooted compared with BAT 881 and MD 23-24. Terminal drought simulation studies in soil tubes indicated that BAT 477 has the ability to grow tap roots under drought conditions, whereas tap root growth was inhibited in DOR 364. Meanwhile, BAT 477 was found to have vigorous lateral root growth without drought stress. This constitutive trait may help it to cope with water deficiency, although the lateral root growth of both genotypes was inhibited under the drought conditions tested. Greenhouse evaluation of 30 RILs of the cross of DOR 364 × BAT 477 using the same method for root phenotyping resulted in identification of two RILs (BT 21138-124-1-4 and BT 21138-6-1-1) with greater ability for fine root development at deeper soil depth than the other RILs tested.

This greenhouse screening technique using soil tubes to determine phenotypic differences in rooting ability under drought stress has been found to be very complementary to field studies to evaluate shoot traits for drought resistance in both parents and advanced lines of common beans.

## Challenges and opportunities for improving common bean for adaptation to drought

Although physiological studies have revealed the role of some traits, especially rooting depth and photosynthate remobilization, the mechanisms behind these traits are not yet defined. Furthermore, the relative importance of other traits is still not understood, for example, the control of stomatal behavior. Nor has the role of metabolites in drought resistance been well studied. In a species as diverse as common beans, and with the potential that it has for introgression from sister species, useful genetic variability may yet be found for other traits and mechanisms that may have a role in drought resistance. Therefore, study of those physiological traits and mechanisms needs to continue. Furthermore, screening conditions to optimize the expression of traits need to be fine-tuned, and the relationship between physiological traits and the QTLs that control them needs to be explored.

While initial QTL studies have been promising, these have mostly been in a limited number of RIL populations, all so far created from crosses within the Middle American genepool (Schneider et al., 1997b; Beebe et al., 2006a). Further studies with populations developed from crosses between genepools or from crosses within the Andean genepool are needed to explore additional diversity for drought resistance QTL alleles, and to analyze the effect of genetic backgrounds on the QTL alleles that have already been identified. As mentioned earlier, there is a need for a larger number of high polymorphism microsatellites to analyze populations derived from intra-genepool crosses. In addition, a highly saturated marker system such as diversity arrays technology (DArT) would be valuable for fine mapping of QTLs. To do this effectively, larger populations are needed for genetic analysis, since most RIL populations in common beans have only been developed with around 100 lines. However, the creation of RIL populations of more than 300 lines presents its own difficulties, given that common beans are a low multiplication species compared to cereals. This also affects the maintenance of RILs.

Alternative population types would also be of interest for the analysis of drought resistance. In this sense, the advanced backcross strategy holds promise for the determination of QTLs that function without the confounding effect of epistasis with alleles from non-commercial sources, since advanced backcross breeding fixes valuable alleles in the genetic background of a commercial parent. If MAS for drought tolerance is to be successful, then understanding the interaction of QTL alleles with multiple genetic backgrounds is important, since breeding programmes usually deal with a range of commercial classes and seed colors representing different genetic backgrounds, genepools and races.

An additional challenge to the genetic understanding of drought resistance is to associate QTLs with their underlying genetic and mechanistic factors, whether these be regulatory genes such as those governing transcription factors, or structural genes such as those involved in hormone pathways, carbon or nitrogen metabolism under drought stress and drought-associated secondary metabolite production. Structural genes for biosynthesis of metabolites such as proline and trehalose would be of interest for common beans, since these two metabolites have had an effect on drought resistance (Farías-Rodríguez et al., 1998; Amede and Schubert, 2003a; Chen, pers. communication; Suárez et al., 2008).

Parts of the abscisic acid (ABA) hormone response pathway would also be sources of candidate genes that may underlie some of the QTLs identified to date, or that are still to be discovered. In addition, candidate genes for carbon accumulation and remobilization from leaves and stems to pods and to seeds such as those encoding sucrose synthase, sucrose-phosphate synthase, and vacuolar or cell wall invertases might also be of interest (Sturm, 1999; Pinheiro et al., 2001). In this regard, the analysis of drought related candidate genes can be an important offshoot of translational genomics that makes use of sequence information in well-studied model species to understand genes that are important to agricultural traits in crop species. Comparative genomics has been exploited to a greater extent in cereals (Bennetzen, 2000) than in legumes, but it may be possible in the future to align QTLs for drought resistance discovered in soybeans (Mian et al., 1996, 1998) or even candidate genes analyzed in this species with their putative orthologous loci in common beans.

Candidate genes important for hormone or metabolite production are relatively straightforward to clone, whereas transcription factors are often members of multigene families that are relatively more difficult to analyze on either a transcriptome or gene-by-gene basis (Udvardi et al., 2007). Furthermore, subtle difference in the expression of transcription factors can have major effects. Therefore, they are difficult to detect with differential display, subtractive library production or array-based analyses (Torres et al., 2006). One example of a transcription factor that is specifically expressed in roots of both tepary beans and common beans was discovered by Rodriguez-Uribe and O'Connell (2006) and is a member of the basic-leucine zipper (bZIP) transcription factor family. Some members of the DREB gene family also appear to be root specific and induced by drought (CIAT, unpublished data). It is important when analyzing candidate genes to base the analysis on the evaluation of gene sequences or expression levels in the best drought resistance source genotypes or species available to the bean researcher.

In terms of breeding, interspecific crosses with *P. acutifolius* in particular continue to be attractive from the standpoint of the very high levels of drought resistance in this species. However, so far, such crosses have been disappointing in terms of what has actually been transferred to common beans. Progress may be facilitated by a better understanding of the mechanisms and traits involved, such that selection in populations may be focused on them. Accessions of *P. acutifolius* are included in physiological studies and, to date, it is evident that deep fine roots are typical of this species and probably contribute to drought resistance. Characterization of *P. acutifolius* accessions for drought resistance should continue to elucidate how its traits and mechanisms can complement those existing in common bean, and to focus selection on the most important and unique traits. At the very least, *P. acutifolius* can serve as a model of how multiple drought resistance traits and mechanisms can combine for high levels of resistance. Interspecific crosses with *P. coccineus* are another opportunity to modify the root structure in potentially useful ways. The root system of *P. coccineus* is much thicker than that of *P. vulgaris* and may be better able to penetrate soil of high bulk density than roots of common beans—a useful trait for compacted soils where root development is limited. This must be confirmed, and the potential value of this trait be evaluated.

In the medium to long term, the challenge will be to match traits and mechanisms to specific environments with regard to patterns of drought (terminal versus intermittent), and associated limitations (e.g., low soil fertility, high temperatures, and local pathogens). This will need to be an iterative process of identifying genetic diversity, defining general classes of traits, and testing these across broad classes of environments. The complexity of this task will defy a strictly rational approach for the foreseeable future, and much will depend on an empirical approach, followed by more cycles of physiological analysis and testing.

Great potential exists for improving drought resistance in common beans. Exploiting this potential will be enhanced by more systematic application of physiological and genomic tools and continued genetic and mechanistic analysis of a range of diverse germplasm both from within the species and from close relatives. At present, the most important traits appear to be those associated with rooting depth and photosynthate remobilization, but other traits may emerge in the future. Effective use of genomic tools will be aided by a better understanding of the physiology of drought response and drought resistance mechanisms. Beans in particular are sensitive to other soil factors, such as compaction or low soil fertility, that will influence the expression of favorable rooting traits. This fact makes the study of drought resistance in beans especially complex, and has important implications for the ultimate expression of drought resistance in farmers' fields. Beans may also be sensitive to environmental factors that influence mobilization of photosynthates to grain. Efficient breeding schemes, managed stress conditions, scaling-up of the use of phenotyping tools, together with genomics and MAS, are expected to improve the efficiency of genetic enhancement for drought resistance in common beans.

### Conflict of interest statement

The authors declare that the research was conducted in the absence of any commercial or financial relationships that could be construed as a potential conflict of interest.

## Footnotes

^1^http://www.regentinstruments.com/
